# Postbiotic Impact on Host Metabolism and Immunity Provides Therapeutic Potential in Metabolic Disease

**DOI:** 10.1210/endrev/bnae025

**Published:** 2024-09-05

**Authors:** Han Fang, Rodrigo Rodrigues e-Lacerda, Nicole G Barra, Dana Kukje Zada, Nazli Robin, Alina Mehra, Jonathan D Schertzer

**Affiliations:** Department of Biochemistry and Biomedical Sciences, Farncombe Family Digestive Health Research Institute, and Centre for Metabolism, Obesity and Diabetes Research, McMaster University, Hamilton, Ontario, Canada, L8N 3Z5; Department of Biochemistry and Biomedical Sciences, Farncombe Family Digestive Health Research Institute, and Centre for Metabolism, Obesity and Diabetes Research, McMaster University, Hamilton, Ontario, Canada, L8N 3Z5; Department of Biochemistry and Biomedical Sciences, Farncombe Family Digestive Health Research Institute, and Centre for Metabolism, Obesity and Diabetes Research, McMaster University, Hamilton, Ontario, Canada, L8N 3Z5; Department of Biochemistry and Biomedical Sciences, Farncombe Family Digestive Health Research Institute, and Centre for Metabolism, Obesity and Diabetes Research, McMaster University, Hamilton, Ontario, Canada, L8N 3Z5; Department of Biochemistry and Biomedical Sciences, Farncombe Family Digestive Health Research Institute, and Centre for Metabolism, Obesity and Diabetes Research, McMaster University, Hamilton, Ontario, Canada, L8N 3Z5; Department of Biochemistry and Biomedical Sciences, Farncombe Family Digestive Health Research Institute, and Centre for Metabolism, Obesity and Diabetes Research, McMaster University, Hamilton, Ontario, Canada, L8N 3Z5; Department of Biochemistry and Biomedical Sciences, Farncombe Family Digestive Health Research Institute, and Centre for Metabolism, Obesity and Diabetes Research, McMaster University, Hamilton, Ontario, Canada, L8N 3Z5

**Keywords:** postbiotics, metabolism, inflammation, metabolic diseases, gut microbiota

## Abstract

The gut microbiota influences aspects of metabolic disease, including tissue inflammation, adiposity, blood glucose, insulin, and endocrine control of metabolism. Prebiotics or probiotics are often sought to combat metabolic disease. However, prebiotics lack specificity and can have deleterious bacterial community effects. Probiotics require live bacteria to find a colonization niche sufficient to influence host immunity or metabolism. Postbiotics encompass bacterial-derived components and molecules, which are well-positioned to alter host immunometabolism without relying on colonization efficiency or causing widespread effects on the existing microbiota. Here, we summarize the potential for beneficial and detrimental effects of specific postbiotics related to metabolic disease and the underlying mechanisms of action. Bacterial cell wall components, such as lipopolysaccharides, muropeptides, lipoteichoic acids and flagellin, have context-dependent effects on host metabolism by engaging specific immune responses. Specific types of postbiotics within broad classes of compounds, such as lipopolysaccharides and muropeptides, can have opposing effects on endocrine control of host metabolism, where certain postbiotics are insulin sensitizers and others promote insulin resistance. Bacterial metabolites, such as short-chain fatty acids, bile acids, lactate, glycerol, succinate, ethanolamine, and ethanol, can be substrates for host metabolism. Postbiotics can fuel host metabolic pathways directly or influence endocrine control of metabolism through immunomodulation or mimicking host-derived hormones. The interaction of postbiotics in the host-microbe relationship should be considered during metabolic inflammation and metabolic disease.

## Graphical Abstract

**Graphical Abstract:**
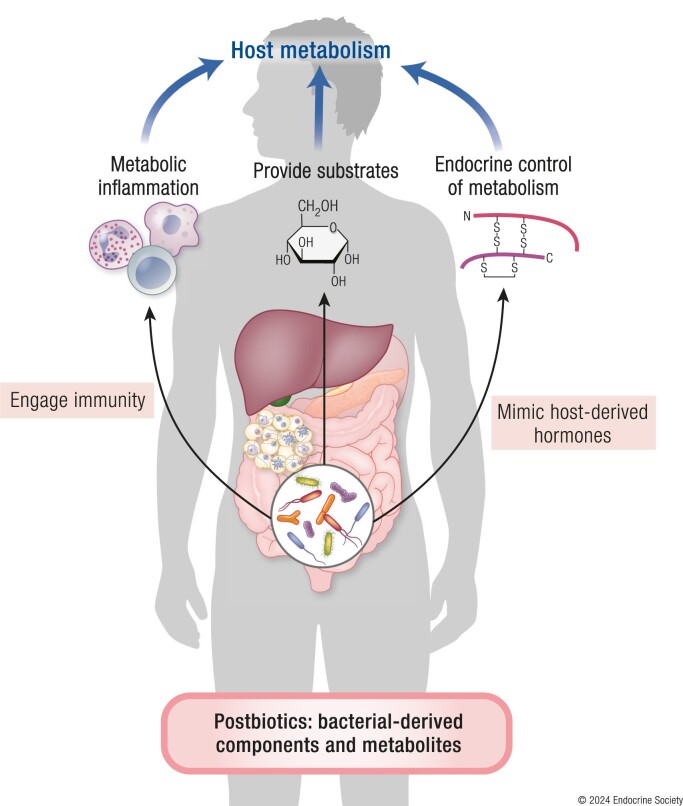

**Essential Points**

- Postbiotics can have context-dependent effects on immunity and metabolism
- Postbiotics can cooperate and have opposing effects on host metabolism
- Postbiotics can provide substrates to directly fuel host metabolic pathways
- Postbiotics can mimic host-derived hormones to regulate endocrine function and metabolism

The gut microbiota is a community of resident microorganisms in the host intestine, which includes bacteria, fungi, viruses, and archaea. The existence of the gut microbiota has been known for centuries and most research has focused on the resident bacteria. In the early 2000s compositional changes in the genetic material of the intestinal bacteria (ie, the microbiome) showed that the Bacteroidetes to Firmicutes ratio is lower in rodent and human obesity (1, 2). The gut microbiota characteristic of obesity was associated with increased capacity for energy harvest and could be a (small) contributor to excess positive energy balance that may promote obesity (3). Causality between gut microbiota and energy metabolism emerged when germ-free mice were colonized with microbiota for only 2 weeks, which equated to increased body fat content (4). Similar findings were observed in germ-free mice that received fecal microbiota transplantation from human twin pairs discordant for obesity, where the obese twin's fecal microbiota promoted a greater increase in fat mass than mice receiving the lean twin's gut microbiota (5). Hence, a transmissible component of adiposity occurs through gut microbiota. In contrast to the relatively fast transmission of adiposity that can happen within a couple of weeks in mouse models, it takes longer for the gut microbiota to transfer some metabolic characteristics that are often associated with obesity, such as endocrine control of glucose metabolism. Over 4 weeks of host exposure time to microbiota derived from diet-induced obese mice is required to promote transmissible insulin resistance despite rapid changes in microbiome (ie, taxonomy) that occur as early as 1 day of feeding obesogenic diet (6). These results highlight the need to move beyond taxonomy and determine the functional units of the gut microbiota that alter specific aspects of host metabolism. The most prolific example of a microbiota-derived factor altering metabolism is lipopolysaccharide (LPS), often called *endotoxin*. LPS is elevated during obesity, which is commonly referred to as *metabolic endotoxemia* (7). Subcutaneous infusion of low levels of LPS via an osmotic minipump promotes hyperglycemia, hyperinsulinemia, and an increase in body and metabolic tissue weight. In line with this finding, germ-free mice monocolonized with the LPS-containing strain Enterobacter cloacae B29 isolated from a patient with morbid obesity developed worse obesity and insulin resistance when fed an obesogenic diet (8).

What is the best approach to target the gut microbiota during obesity and metabolic disease? Identification of bacterial strains or small communities of strains may foster development of probiotics. Co-housing germ-free mice colonized with gut microbiota from human twins who are discordant for obesity showed that the domination of transmissible leanness correlated with invasion of specific members of Bacteroidetes from lean gut microbiota (5). It is possible that improved mining of the microbiome may yield strains of bacteria that can work together to promote metabolic health. However, an important consideration is whether live bacteria (ie, probiotics) or altering live bacterial community (ie, prebiotics) is required or the best approach to influence host metabolism.

##  

### Prebiotics and Probiotics

The gut microbiota can be targeted by intake of prebiotics, or probiotics, or the combination of prebiotics and probiotics, also known as *synbiotics*. Probiotics are defined as live microorganisms that have health benefits. Prebiotics are defined as nondigestible food ingredients that promote the growth of microorganisms that have health benefits. A number of studies have shown improvements in aspects of metabolic disease using probiotics, prebiotics, and synbiotics in preclinical animal models, but the efficacy of any of these approaches in human clinical studies is unclear (9). In addition, safety is a concern given that live microorganism ingestion or manipulation may cause horizontal gene transfer and the potential for pathogen expansion and/or infection. For instance, the transfer of antibiotic resistance genes may lead to increased antibiotic resistance. Probiotics are also difficult to produce in a way that delivers the same effective dose, given the need for standardization of bacterial growth, consistently achieving the same dose after production and transport, and the possibility for evolution of the bacterial strain(s). Moreover, certain bacterial strains require rigorous growth and maintenance conditions (eg, sensitivity to oxygen), presenting logistical problems with transportation and storage. Prebiotics lack specificity and it is not yet clear how prebiotics will be developed to specifically promote the growth of beneficial strains in highly variable communities of bacteria between individuals and even within individuals over time. Fecal microbiota transplantation has also been explored as a potential therapy for metabolic diseases, but this approach has many of the same limitations as probiotics and prebiotics. One of the major issues of these prebiotic and probiotic approaches is lack of mechanisms of action directly implicating the host-microbe responses that underpin changes in host metabolism.

### Postbiotics

Postbiotics are defined as “preparation of inanimate microorganisms and/or their components that confers a health benefit on the host” by the International Scientific Association for Probiotics and Prebiotics (ISAPP) (10). However, this definition is limited to a health benefit and does not include the potential detrimental effects of postbiotics. This definition also does not consider context-dependent effects and interactions of microbial molecules, which can dictate their function, including beneficial effects and deleterious effects for various aspects of health. In our opinion, postbiotics can be sorted into the following 2 categories: (i) bacterial components and (ii) bacterial metabolites. In a few pioneer studies, postbiotics have shown promise in mitigating specific aspects of metabolic disease. *Akkermansia muciniphila* is a mucin-degrading bacterium in the human intestinal tract and its abundance is negatively correlated with obesity and type 2 diabetes (T2D) (11). As a probiotic, administration of *A. muciniphila* lowered fat mass, metabolic endotoxemia, adipose tissue inflammation, and insulin resistance in diet-induced obese mice (11). Intriguingly, pasteurized *A. muciniphila* has a greater capacity to lower fat mass and insulin resistance. It is not yet clear why the pasteurized bacterium has a greater effect, but it may be linked to increased whole-body energy expenditure and fecal energy excretion (12, 13). These results suggest that the beneficial effects of *A. muciniphila* on glucose and energy metabolism are mediated by its postbiotics. Then, a more refined postbiotic was developed using the protein Amuc_1100, isolated from the outer membrane of *A. muciniphila,* which partly recapitulated the metabolic improvements from the whole (pasteurized) bacterium. This exemplar demonstrates the potential for postbiotics and suggests that multiple postbiotics derived from the whole bacterium cooperate to influence host metabolism. In fact, the totality of the entire intestinal microbiota, when sterilized/pasteurized and injected subcutaneously at the correct dose can improve endocrine/insulin control of glucose metabolism in lean, obese and prediabetic mice (14, 15). Multiple labs have shown that the net effect of subcutaneously injecting all postbiotics prepared from an upper gut bacterial extract improved glucose and insulin tolerance in both male and female mice with or without obesity (14, 15). This subcutaneous “vaccination” with all postbiotics required both innate and adaptive immunity to confer improvements in blood glucose control. Bacterial cell wall sensing by the innate immune system was required for a postbiotic vaccination to improve blood glucose (14). The adaptive immune system was sufficient to convey the effects of bacterial vaccination since naïve mice are protected from high-fat diet (HFD)-induced dysglycemia and insulin resistance after receiving immune cells transfer from gut bacterial extract treated obese mice (15). However, it is not known which postbiotics cooperate (or limit) changes in host metabolism.

Postbiotics do not require the use of live bacteria, avoid horizontal gene transfer with commensal bacteria (and the potential for antibiotic resistance), and are positioned to be easier to store, transport and standardize. Therefore, the scope of this review is to summarize current knowledge on the impact of postbiotics on host metabolism, immunity, and endocrine function related to metabolism, specifically glucose and insulin metabolism related to obesity. We will discuss specific cellular components and metabolites of bacteria that alter host metabolism and immunity. We will discuss mechanisms of action where, in general, bacterial components engage immune responses that alter endocrine control of metabolism and bacterial metabolites can act as substrates for host metabolism in addition to influencing immunity (Fig. 1).

**Figure 1.**
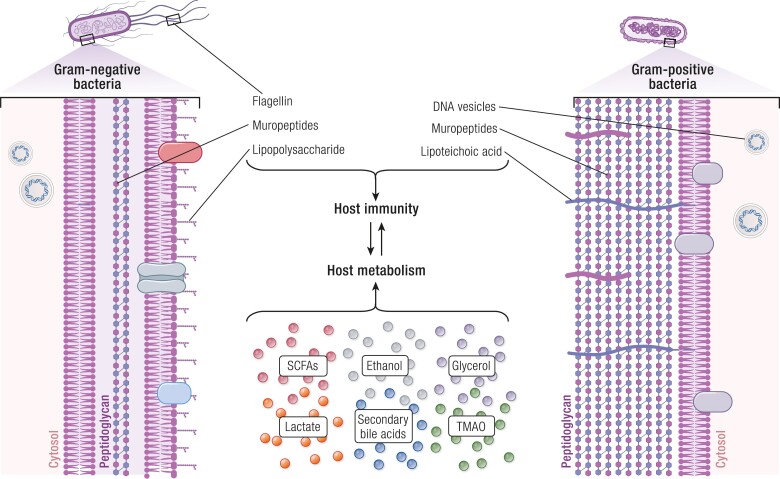
Postbiotics derived from both Gram-negative and Gram-positive bacteria in the gut modulate host immunity and metabolism. Gram-negative (left) and Gram-positive (right) bacteria generate unique and common postbiotics. In general, Gram-negative bacteria have cell wall component–based postbiotics that include lipopolysaccharides, specific muropeptides within peptidoglycan, flagellin in the flagellar filament of flagellated bacteria, and DNA vesicles that can be exocytosed into the intestinal lumen. Gram-positive bacteria also have DNA vesicles and muropeptides, in addition to lipoteichoic acid incorporated into the peptidoglycan layer. The muropeptides in Gram-positive bacteria can have a different structure and immunogenicity from those in Gram-negative bacteria. All these postbiotics can engage host immunity, and consequently influence host metabolism. Furthermore, host metabolism can be directly modulated by factors secreted by both types of bacteria, such as short-chain fatty acids (SCFAs), ethanol, glycerol, lactate, secondary bile acids, and trimethylamine oxide (TMAO).

## Postbiotics: Bacterial Components

### Lipopolysaccharides

LPS is a component of the outer membrane of the cell wall of Gram-negative bacteria. LPS is made of an O-specific antigen, a core oligosaccharide, and a multi-acylated lipid A (16). LPS is a well-known trigger of inflammation and metabolic dysfunction by inducing the production of proinflammatory cytokines, such as tumor necrosis factor alpha (TNF-α), interleukin-6 (IL-6) and IL-1β (7). Cani et al reported that 4 weeks of HFD feeding increased circulating LPS (7). LPS is not just a biomarker of metabolic inflammation, rather LPS contributes to obesity-induced inflammation and insulin resistance (7). Fei and Zhao showed that the introduction of an endotoxin-producing bacterium, Enterobacter cloacae strain B29, from obese humans caused obesity and insulin resistance in germ-free mice (8). LPS absorption is significantly increased in the small intestine after ingestion of dietary fat or an obesogenic diet (17-20). Canonically, LPS is thought to be incorporated into chylomicrons in the Golgi apparatus and then absorbed by adipocytes and macrophages in adipose tissue and cleared by Kupffer cells in the liver (20). However, recent in vivo evidence confirms that LPS directly enters the liver through portal circulation after crossing the small intestine barrier via lipid raft- and cluster of differentiation 36 (CD36)-mediated transport mechanisms (17). It appears that only a small portion of LPS is absorbed through the chylomicron pathway and increased paracellular transport of LPS (17).

LPS engages toll-like receptor (TLR)4 and cluster of differentiation 14 (CD14)-mediated immune responses including toll/IL-1 receptor domain-containing adaptor protein (TIR) and myeloid differentiation factor 88 adaptor protein (MyD88) signaling cascades that promote proinflammatory responses (21). These proinflammatory responses can cause dysmetabolism through many mechanisms of action, including the generation of cytokines and priming of other immune response such as the NOD-like receptor family pyrin domain-containing 3 (NLRP3) inflammasome, which promotes insulin resistance and dysglycemia (21). Deletion of components of TLR4 signaling influences metabolic inflammation, such as CD14 deletion in genetically obese (ob/ob) mice, which lowered markers of inflammation in the visceral and subcutaneous fat (22). In addition, mice with defective CD14 signaling are protected from LPS and HFD-induced glucose intolerance and inflammation (7).

LPS also influences endocrine factors. Daily intraperitoneal injection of 100 μg/kg LPS for 2 weeks in Zucker rats fed a diet rich in disaccharides led to decreased plasma adiponectin levels, increased plasma leptin levels, and increased hepatic steatosis alongside increased markers of liver lipogenesis and inflammation indicative of metabolic dysfunction-associated fatty liver disease (MAFLD) (23). LPS also interacts with macronutrients to promote gut barrier dysfunction and hepatic steatosis. For example, fructose ingestion cooperates with microbiota-derived LPS to compromise gut barrier function and allows penetration of LPS into the hepatic environment where it promotes resident macrophage-induced inflammation and hepatocyte lipogenesis (24).

**Figure 2.**
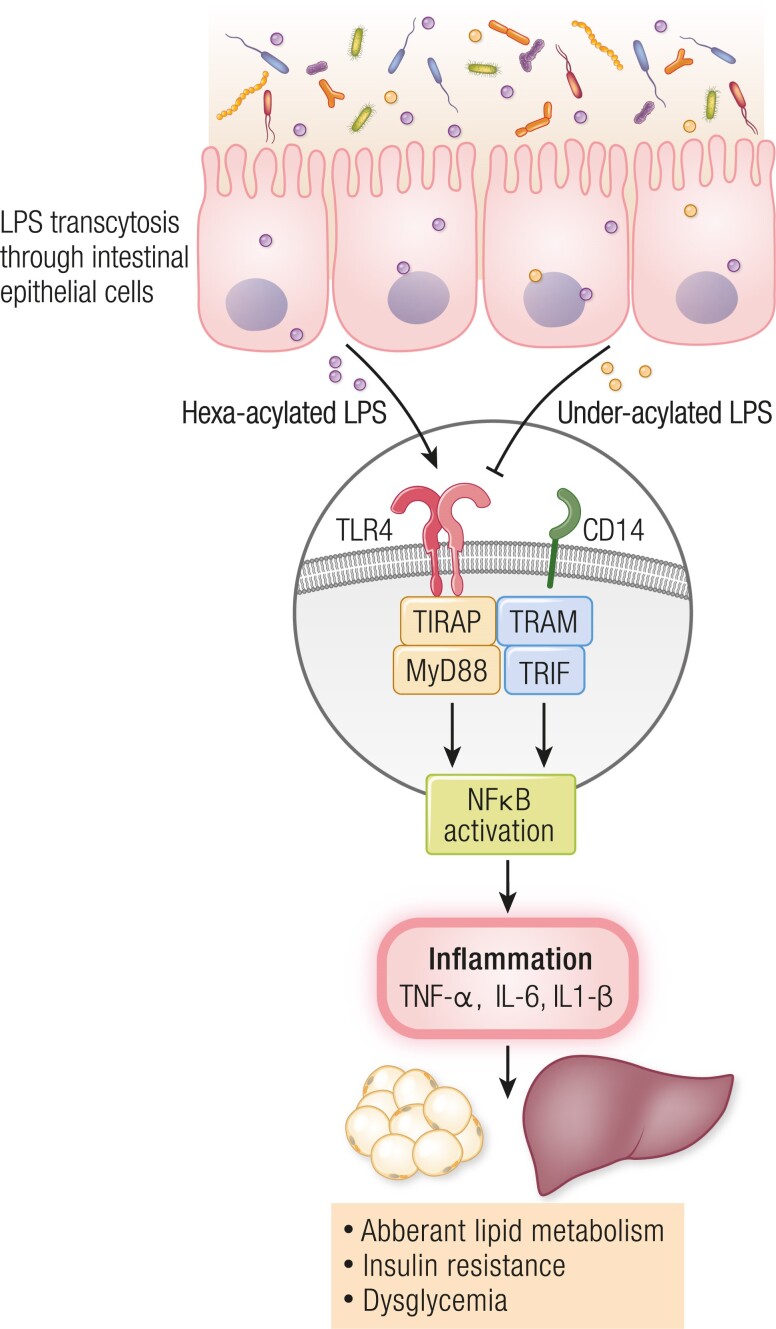
Different types of LPS promote deleterious or beneficial metabolic endotoxemia. Gut microbiota-derived LPS can penetrate the gut mucosal barrier through intestinal epithelial cells transcytosis, which is increased after ingestion of an obesogenic diet. Hexa-acylated LPS (eg, from strains of *E. coli*) causes metabolic inflammation by activating TLR4 and downstream NF-κB signaling, promoting inflammation, dysglycemia, insulin resistance, and lipid accumulation. Under-acylated LPS (eg, from strains of *R. sphaeroides*) is an antagonist of TLR4, which mitigates metabolic inflammation and can attenuate the metabolic inflammation caused by hexa-acylated LPS and other inflammatory triggers during obesity. Hence, the type of LPS derived from different strains of bacteria dictates whether metabolic endotoxemia is deleterious or beneficial. Abbreviations: CD14, cluster of differentiation 14; IL-1β, interleukin 1 beta; IL-6, interleukin 6; LPS, lipopolysaccharide; MyD88, myeloid differentiation factor 88; NF-κB, nuclear factor kappa B; TIRAP, toll/interleukin 1 receptor domain-containing adaptor protein; TLR4, toll-like receptor 4; TNF-α, tumor necrosis factor alpha; TRAM, TRIF-related adaptor molecule; TRIF, TIR-domain-containing adaptor-inducing beta interferon.

Nearly all research on metabolic inflammation has focused on LPS that activates TLR4, such as LPS derived from specific *Escherichia coli* strains of bacteria. However, many different strains of bacteria produce many types of LPS that have unique structural and biochemical features. LPS from different species and strains of bacteria can activate or antagonize TLR4 (25, 26). The initial concept of metabolic endotoxemia and LPS promoting metabolic dysfunction did not account for bacterial strain-specific variations in LPS structure. Typically, LPS derived from *E. coli* which has hexa-acylated lipid A was used in metabolism studies. While a hexa-acylated LPS from *E. coli* activates TLR4, penta-acylated LPS can dose-dependently antagonize TLR4 activation (27). Anhê et al showed that intraperitoneal injection of penta-acylated (ie, under-acylated) LPS, such as LPS derived from *Rhodobacter sphaeroides* counteracts dysglycemia caused by *E. coli* LPS (28). Further, subcutaneous delivery of *R. sphaeroides* LPS in osmotic minipumps improved blood glucose control, lowering blood insulin and adipose tissue inflammation in obese mice (28). Penta-acylated LPS derived from *R. sphaeroides* can directly oppose deleterious changes in gut barrier function, adipose tissue inflammation, insulinogenic potential and blood glucose control caused by *E. coli* LPS (28). Therefore, under-acylated LPS is a postbiotic that generates metabolically beneficial endotoxemia (Fig. 2).

### Muropeptides

Peptidoglycan is a critical component of the bacterial cell wall which maintains the bacteria structural integrity. It consists of chains of alternating sugars (N-acetylglucosamine and N-acetylmuramic acid) and amino acids. Muropeptides are the building blocks of peptidoglycan, and they are recycled by both Gram-positive and Gram-negative bacteria during cell division. Muropeptides are constantly released from bacteria by escaping peptidoglycan recycling or enzyme-induced peptidoglycan degradation (29). Muropeptides are recognized by intracellular pattern recognition receptors, including nucleotide oligomerization domain proteins 1 and 2 (NOD1 and NOD2). NOD1 and NOD2 are expressed in professional immune cells such as macrophages, and parenchymal cells in metabolically active tissues such as liver, fat, and muscle (30). A dipeptide, gamma-D-glutamyl-meso-diaminopimelic acid (iE-DAP), is the minimal core structure that can be recognized by NOD1 (31). Other synthetic molecules, including L-Ala-γ-D-Glu-mDAP (Tri-DAP), FK156, and FK565, that mimic iE-DAP can also act as ligands for NOD1 (32, 33). In general, postbiotics that activate NOD1 activate a proinflammatory nuclear factor kappa-light-chain-enhancer of activated B cells (NF-κB) cascade, which promotes metabolic inflammation and insulin resistance. NOD1 ligands also act directly on adipocytes to increase proinflammatory chemokines and cytokines such as C-X-C motif chemokine ligand (CXCL) 1, CXCL10, monocyte chemoattractant protein 1 (MCP-1), RANTES, TNF-α, and IL-6 (32-36). NOD1 activity is increased in subcutaneous adipose tissue from people with obesity/metabolic syndrome, which is correlated with increased NF-κB activity and serum levels of IL-6 and MCP-1 (35). Postbiotics that activate NOD1 (such as Tri-DAP and iE-DAP) lower insulin-stimulated glucose uptake and promote insulin resistance by increasing stress kinase activation thereby promoting insulin receptor substrate 1 (IRS-1) serine phosphorylation and disrupting insulin signaling in adipocytes (32, 34). NOD1 ligands are potent stimulators of adipose tissue lipolysis mediated by ERK, PKA, and NF-κB (37). Postbiotics that activate NOD1 also cause cell-autonomous inflammation and insulin resistance in hepatocytes (33).

**Figure 3.**
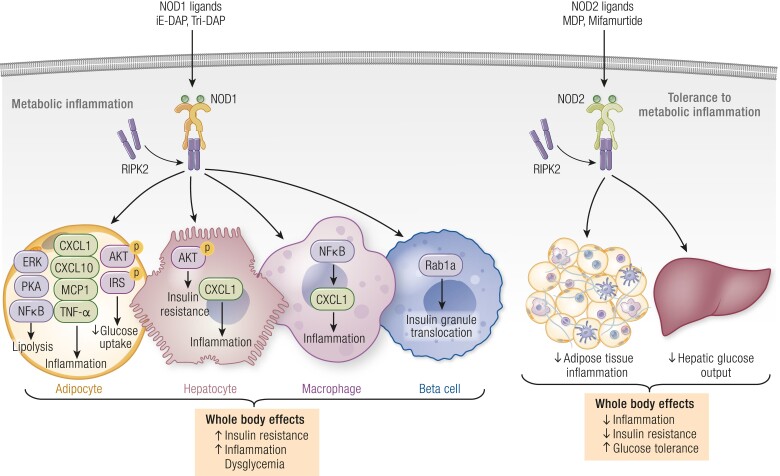
Different types of muropeptides have opposing effects on metabolic inflammation and glucose homeostasis. Muropeptides from certain bacteria (usually Gram-negative bacteria) that activate NOD1 such as γ-D-glutamyl-meso-diaminopimelic acid (iE-DAP) or L-Ala-γ-D-Glu-mDAP (Tri-Dap) cause metabolic inflammation and dysglycemia. Muropeptides from certain bacteria (usually more abundant in Gram-positive bacteria) such as muramyl dipeptide (MDP), which activate NOD2, mitigate metabolic inflammation and dysglycemia. NOD2 can also be activated by the orphan drug Mifamurtide. Both NOD1 and NOD2 recruit RIPK2 to propagate downstream effects on immunity and metabolism. In adipocytes, hepatocytes, and macrophages, NOD1 activation leads to increased proinflammatory gene (*Cxcl1*, *Cxcl10*, *Mcp1*, and *Tnfa*) expression, increased lipolysis that is regulated by ERK/PKA/NF-kB signaling, and decreased glucose uptake by disrupting insulin signaling. In pancreatic beta cells, NOD1 activation recruits RIPK2 and Rab1a to regulate insulin vesicle translocation. Overall, NOD1 ligands promote insulin resistance, dysglycemia, and inflammation in vivo. Repeated NOD2 activation leads to lower obesity-induced inflammation, lower insulin resistance, and improved glucose control, which includes lower hepatic glucose output. Repeated exposure to NOD2 ligands lowers adipose tissue inflammation and induces immunological tolerance to stressors that cause metabolic inflammation and insulin resistance, including obesogenic diets. Abbreviations: CXCL1 and 10, C-X-C motif chemokine ligand 1 and 10; IRS-1, insulin receptor substrate 1; MCP-1, monocyte chemoattractant protein-1; MDP, muramyl dipeptide; NF-κB, nuclear factor kappa B; NOD1 and NOD2, nucleotide oligomerization domain proteins 1 and 2; RIPK2, receptor-interacting serine/threonine-protein kinase 2; TNF-α, tumor necrosis factor alpha.

Receptor-interacting serine/threonine-protein kinase 2 (RIPK2) is known to promote activation of mitogen-activated protein kinase and NF-κB and signaling after NOD1 activation by muropeptides (38, 39). RIPK2 is required in NOD1-activating muropeptides-induced inflammation, and lipolysis in adipose tissue (40). In pancreatic beta cells, NOD1 ligands activate NOD1 and recruit RIPK2 and Rab1a to modulate insulin vesicle distribution (41). The totality of the tissue-specific effects of NOD1 activation leads to whole-body insulin resistance, dysglycemia, and metabolic inflammation, all of which require RIPK2 (Fig. 3) (33, 41, 42). This has prompted investigation of RIPK2 inhibitors in metabolic disease. However, RIPK2 is also required for the action of postbiotics that activate NOD2.

Muramyl dipeptide (MDP) is the minimal bioactive muropeptide motif and a ligand for NOD2. In skeletal muscle myotubes, MDP treatment activates the inhibitor of nuclear factor-kβ kinase/NF-kB and c-Jun N-terminal kinases signaling pathways, leading to upregulation of proinflammatory cytokine gene expression. MDP-induced proinflammatory response results in impaired insulin signaling and lower insulin-stimulated GLUT4 translocation, consequently lowering insulin-stimulated glucose uptake in muscle cells (30). Despite the adverse effects of MDP during in vitro experiments, the net effect of NOD2 activation in vivo is insulin sensitizing (in mice). MDP intraperitoneal injection improves blood glucose control and lowers insulin resistance in various models of obesity and metabolic endotoxemia (43). The beneficial effects of NOD2-activating postbiotics are independent of diet or microbiome composition. MDP intraperitoneal injection also improves adipose tissue inflammation by lowering proinflammatory cytokines and chemokines, which requires nonhematopoietic RIPK2 (Fig. 3) (43, 44). Moreover, interferon regulatory factor 4 (IRF4) plays a crucial role in the ability of MDP to reduce insulin resistance and metabolic inflammation through activation of NOD2. It is not yet clear if MDP-NOD2 is a druggable postbiotic pathway in humans with obesity or metabolic disease. Mifamurtide is a synthetic orphan drug that mimics MDP and activates NOD2, which has been used to treat osteosarcoma. The MDP-mimetic effects of mifamurtide have been tested in mice, where intraperitoneal injection mifamurtide is an insulin sensitizer and lowers blood glucose during metabolic endotoxemia (43). This highlights the importance of discovering the mechanisms of action of postbiotics. In addition to the opposing effects of NOD1 and NOD2 postbiotics on metabolism, there are cell-specific effects. More information on the mechanisms of action is needed, since the key cell type for IRF4-driven changes in immunity vs metabolism and sex differences of the postbiotic actions of MDP are not yet clear (Fig. 3). This may foster the development of refined synthetic postbiotics or use of postbiotic combinations that target NOD2 and promote beneficial metabolic endotoxemia using LPS that antagonizes TLR4.

### Flagellin

Flagellin is part of the bacterial locomotor appendage flagellum used in motility (45). Flagellin can penetrate the intestinal mucosal barrier and enter the host circulation (46). Flagellin engages TLR5 to cause context-dependent changes in immunity that can promote or suppress inflammation (45). While traditionally viewed as a virulence factor, flagellin is an immunomodulatory factor that can promote or mitigate tissue-specific metabolic dysfunction depending on the site of action and dose of flagellin (45-47). In vitro experiments demonstrate that flagellin can induce pancreatic beta cell dysfunction by promoting inflammation, impairing insulin gene expression, causing acute hypersecretion of insulin, and reducing insulin content in cultured islets (46). These data are consistent with weekly in vivo intraperitoneal injections of flagellin in mice, which promotes hyperinsulinemia, a marker of beta cell dysfunction (46). It is not yet clear how endogenous flagellin derived from the microbiota influences host metabolism, but circulating flagellin levels positively correlate with glycated hemoglobin (HbA1c) levels in people with T2D (46).

Despite the effects of flagellin on pancreatic beta cells, there is evidence that administration of this postbiotic protects against aspects of metabolic disease. Obesity is associated with increased levels of fecal flagellin, which is hypothesized to reflect a bacterial community with enriched with motile bacteria that can penetrate the intestinal mucus layer resulting in microbial encroachment and host inflammation (47). Host defense mechanisms are supposed to coat intestinal bacteria with flagellin-specific IgA antibodies to protect against encroachment (48). However, the increased bacteria encroachment in metabolic disease may be linked to lower-than-expected flagellin-IgA relative to the number of flagellated bacteria present in the gut. Indeed, people with overweight or obesity have lower flagellin-specific IgA and higher flagellin in the feces (47). To boost immunoglobulins targeting flagellin, repeated flagellin intraperitoneal injections in mice elicited a mucosal flagellin-specific IgA antibody response that can alter the intestinal bacterial profile, reducing flagellin expression to prevent microbial encroachment, lower inflammation, and protect against diet-induced obesity (47). These mice that were “immunized” with intraperitoneal injections of flagellin also gained less weight and had reduced adiposity and intestinal inflammation (47). In addition, targeting hepatic TLR5 signaling to treat liver fibrosis using postbiotics like flagellin has also been shown. Intraperitoneal flagellin injections in mouse models of hepatic fibrosis significantly reduced the severity of fibrosis through TLR5 and type 1 interferon responses (49). These data suggest that flagellin is a postbiotic that has context-dependent and tissue-specific effects, where separating the endocrine actions in the pancreas vs those in metabolic tissues and the gut is a key future goal.

### Lipoteichoic Acids

Lipoteichoic acids (LTAs) are amphiphilic macromolecules that contain hydrophobic and hydrophilic components in the cell wall of Gram-positive bacteria (50). LTAs provide growth, stability, and protection against cationic antimicrobial peptides. Among taxonomic groups, LTAs have wide structural diversity and can act as virulence factors or immunomodulatory agents (51). As microbe-associated molecular patterns, LTAs are recognized by host pattern recognition receptors, but these bacterial components can also modulate host metabolism and can even mitigate aspects of metabolic disease (52, 53). Oral LTA supplementation with heat-killed *Lactobacillus paracasei* strain D3-5 lowered glucose intolerance, insulin resistance, and hepatic steatosis and adipocyte size in diet-induced obese mice (52). LTA supplementation via oral gavage also lowered markers of metabolic inflammation including the number of adipose tissue crown-like structures in obese mice (52). These metabolic benefits were associated with lower intestinal colonic inflammatory markers and lowered gut permeability due to enhanced mucin production (52). Oral administration of LTA altered the composition of the microbiome and increased the relative abundance of the mucin degrader *Akkermansia muciniphila*. In vitro and in vivo results demonstrate that LTA worked in a unique way compared to peptidoglycan, where LTA stimulated goblet cell mass and mucin production in goblet cells through activation of TLR2 (52). Multiple sources of LTA appear promising to confer metabolic benefits, since supplementing LTA from *Bifidobacterium animalis* subsp. *lactis* CECT 8145 (BPL1) showed that LTA lowered fat deposition through the insulin-like growth factor-1 signaling pathway in *Caenohabditis elegans* (53). Overall, these results demonstrate that LTA from multiple bacterial sources can lower adiposity and mitigate metabolic inflammation in animal models.

### Bacterial Vesicles That Contain DNA

Increased gut permeability associated with obesity generates a permissive environment that allows microbial products, such as microbial DNA, to translocate from the intestinal lumen to host metabolic tissues (54). Many different microbial species and strains can produce extracellular vesicles that contain nucleic acids, including DNA, which can subvert intestinal barriers and enter the host circulation (50). Once circulating, these vesicles can accumulate in metabolic tissues, such as pancreatic beta cells and hepatocytes, to promote inflammation and dysmetabolism (55, 56). In general, blood and tissue macrophage subsets can clear bacterial vesicles and their cargo through the complement cascade, which in lean individuals prevents excessive accumulation of bacterial DNA in metabolic tissues. However, obesity lowers the frequency of macrophage subsets that can remove vesicles leading to increased bacterial DNA in metabolic tissues, which promotes insulin resistance (55-57).

People with obesity and T2D, as well as obese HFD-fed mice, have higher levels of bacterial DNA containing vesicles in circulation and within the pancreatic beta cells (55). This is also seen in hepatocytes and hepatic stellate cells of people with MAFLD and metabolic dysfunction-associated steatohepatitis (MASH) and mice fed an obesogenic Western-style diet (56). Adoptive transfer of bacterial extracellular vesicles containing microbial DNA is sufficient to promote metabolic dysfunction in mice, including glucose intolerance, insulin resistance and increased hepatic glucose output (55-57). Furthermore, testing vesicles from obese germ-free mice showed that vesicles must contain microbial products to alter host metabolism (55). Bacterial DNA deposited within metabolic tissues activates the cGAS/STING pathway promoting inflammation, which impairs insulin production and lowers insulin secretion from pancreatic islets in addition to promoting hepatic steatosis, inflammation, and fibrosis (55, 56). In vitro exposure to bacterial vesicles containing bacterial DNA promotes insulin resistance in cultured adipocytes and hepatocytes (57). Both islet and hepatic Vsig4+ macrophages prevented infiltration (or promoted clearance) of intestinal-derived vesicles containing bacterial DNA through complement C3-mediated opsonization; however, obesity-related lowering of these macrophages leads to bacterial DNA accumulation within these metabolic tissues (55, 56). Altogether, extracellular vesicles can transport bacterial DNA to metabolic tissues to influence host inflammation and metabolism. Bacterial vesicles contain other cargo, but it is not yet clear how all the postbiotics in vesicles cooperate to alter host metabolism.

## Postbiotics: Metabolites

### Short-Chain Fatty Acids

Fermentation of nondigestible carbohydrates by the microbiota can result in production of short-chain fatty acids (SCFAs), such as acetate, propionate, and butyrate. They are the key energy sources for intestinal epithelial cell proliferation and differentiation. The role of SCFAs in regulating energy homeostasis and glucose and lipid metabolism have been extensively studied in the past few decades, and are thoroughly reviewed (58-61). Therefore, we will only highlight several key points related to the metabolic and immune actions of acetate, propionate, and butyrate acting as postbiotics. In rodents, acetate, propionate, and butyrate all increase anorexic hormones such as glucagon-like peptide 1 (GLP-1) and plasma peptide YY (PYY) mediated by free fatty acid receptor 2 (FFAR2, also termed G-protein coupled receptor 43), which suppresses orexigenic neuropeptide Y neuron activity in the hypothalamus, resulting in decreased food intake and subsequent weight loss via a gut-brain axis (62-65). All 3 of these SCFAs also increase energy expenditure in diet-induced obese mice (63, 66, 67). Similar findings are found in human studies. Long-term propionate supplementation specifically to the colon by giving an inulin-propionate ester significantly reduces weight gain and liver fat in people that are overweight (68). Acute acetate and inulin-propionate ester colonic infusion increases PYY and GLP-1 concentrations and fat oxidation and also reduces energy intake in people that are overweight or obese (68, 69). However, there are conflicting results for the role of acetate and food intake in obesity since acetate activates the parasympathetic nervous system, promoting ghrelin secretion and hyperphagia, which promotes obesity and the consequent obesity-associated metabolic dysfunction (70).

SCFAs have been proposed as a mechanism of action for prebiotics such as inulin. However, acute increases in SCFAs after a large dose of inulin ingestion does not affect PYY and GLP-1 secretion in people that are lean or living with obesity (71). It is noteworthy that the inulin-induced increase in circulating propionate and acetate were lower than direct delivery of propionate and acetate in other clinical studies. Hence, inulin-induced increases in SCFAs may have not reached the threshold required for activating PYY and GLP-1. Beyond effects on food intake, SCFAs can also affect basal metabolism and substrate selection. Acetate, propionate, and butyrate induce a metabolic shift from lipogenesis to fatty acid oxidation in adipose and liver tissue by downregulating peroxisome proliferator-activated receptor gamma and subsequent activation of uncoupling protein 2-AMP-activated protein kinase (AMPK)-acetyl-CoA carboxylase signaling pathway (67). This activation of AMPK leads to an increase in hepatic lipid oxidation, thereby lowering hepatic fat accumulation in preclinical models of fatty liver disease (63, 67, 72-75). Additionally, oral butyrate administration through intragastric gavage or dietary supplementation increases thermogenesis in brown adipose tissue through gut-brain neural circuit and fatty acid oxidation in skeletal muscle (63, 66).

In addition to actions on energy and lipid metabolism, SCFAs can influence glucose metabolism. Butyrate and propionate can activate intestinal gluconeogenesis, which is an integrative signal to the brain that can relay responses that confer metabolic benefits such as lower food intake and lower systemic blood glucose levels (76). SCFAs can also improve blood glucose control by altering endocrine control of metabolism, including insulin levels. Acetate potentiates glucose-stimulated insulin secretion through activation of the G-protein coupled signaling and FFAR2 in pancreatic beta cells (77). Studies that investigate the role of SCFAs (mainly butyrate) in regulating immune system and inflammatory responses mainly focused on diseases in the gut such as inflammatory bowel syndrome and colon cancer (78, 79). To date, there is insufficient evidence to clearly define how SCFAs alter inflammatory responses in the context of metabolic diseases. Acetate, propionate, and butyrate have been shown to lower LPS-induced production of proinflammatory factors, including TNF, IL-1β, IL-6, and IFNgamma in vitro (80, 81). Tributyrin, a precursor of butyrate, has been reported to reduce adipose tissue inflammation through the activation of G-protein coupled receptor 109a in diet-induced obese mice (82). More studies are needed to fully explore the function of SCFAs in regulating inflammatory profile in obesity and metabolic disease.

It is noteworthy that SCFAs can be part of other microbial metabolites. For example, the propionate derivative imidazole propionate, which is a microbial metabolite of histidine, has been shown to promote dysglycemia and prevent the glucose lowering benefits of metformin by activating mTORC1 and suppressing insulin signaling in hepatocytes (83, 84). Imidazole propionate is increased in the circulation of people with T2D (83, 84). Future work could focus on understanding how and why the microbiota produces SCFAs vs SCFA-derivatives that can have opposing functions on host metabolism.

### Secondary Bile Acids

**Figure 4.**
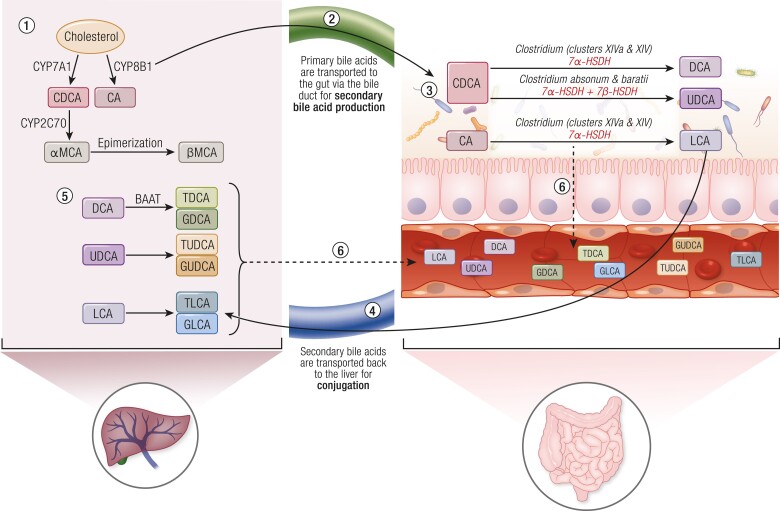
Secondary bile acids are produced by bacterial species in the intestines. (1) Primary bile acids, CA and CDCA, are produced in the liver during cholesterol metabolism. In mice, 2 additional primary bile acids are produced, namely alpha- and beta-MCA. (2) These primary bile acids are then transported to the gut, (3) where they are metabolized by 7a-HSDH in bacteria to produce secondary bile acids, DCA and LCA, whereas UDCA is produced by the activity of both 7a- and 7b-HSDH. (4) These secondary bile acids are transported back to the liver, (5) where they are conjugated by liver enzyme, BAAT. (6) Conjugated and unconjugated bile acids are absorbed into the bloodstream. Abbreviations: 7a-HSDH, 7a-hydroxysteroid dehydrogenase; 7b-HSDH, 7b-hydroxysteroid dehydrogenase; BAAT, bile acid co-enzyme A:amino acid *N*-acyltransferase; CA, cholic acid; CDCA, chenodeoxycholic acid; DCA, deoxycholic acid; LCA, lithocholic acid; MCA, muricholic acid.

Bile acids (BAs) are naturally produced emulsifiers that aid in the absorption of lipids and certain vitamins from the gut into the bloodstream. BAs are produced in the liver through the breakdown of cholesterol into more amphipathic species (85). Once in the gut, ∼95% of BAs are absorbed and delivered back to the liver (86). However, a fraction of BAs remains in the gut and move into the colon where they pose a threat to bacterial survival due to their high acidity (86). Therefore, gut bacteria modify these primary BAs through a series of enzymatic reactions to produce more tolerable substances, secondary BAs (86). Different species and strains of bacteria catalyze the production of a diverse repertoire of secondary BAs through dehydroxylation, oxidation, and epimerization (Fig. 4) (87, 88). Primary BAs undergo 2 main steps: first, deconjugation catalyzed by bacterial bile salt hydrolase activity, followed by 7-alpha-dehydroxylation catalyzed by a series of enzymatic reactions (89). This results in the conversion of primary BAs cholic acid and chenodeoxycholic acid into secondary BAs deoxycholic acid and lithocholic acid (89). These secondary BAs can be further conjugated to produce taurodeoxycholic acid (TDCA), glycodeoxycholic acid (GDCA), and ursodeoxycholic acid (UDCA) as well as taurolithocholic acid and glycolithocholic acid (GLCA), known as tertiary BAs. These microbially produced secondary BAs and the resultant tertiary species are then reabsorbed into the bloodstream (87, 88). BAs are ligands of G-protein coupled bile acid receptor 5 (TGR5) which modulates energy balance, endocrine control of metabolism (ie, insulin levels and insulin action), and can influence immune responses. Secondary BAs also engage farnesoid X receptor (FXR) which regulates metabolism of BAs, glucose, and lipids (90, 91).

It is not yet clear which BAs mediate specific host metabolic outcomes related to characteristics of metabolic disease, but a few concepts may lead to postbiotic approaches related to BAs. In general, liver metabolic dysfunction is associated with higher levels of many circulating BAs, especially secondary BAs (92). Diet-induced obese mice have higher levels of bile salt hydrolases, a class of bacterial enzymes involved in the conversion of primary to secondary BAs (92). Although the role of BAs in the progression from obesity to MAFLD and MASH is not yet clear, higher 12-alpha-OH BAs is a consistent marker of MASH since 12-alpha-OH BAs levels were higher in 3 mouse models of MASH and in patients with MASH and liver fibrosis (92). The conjugated secondary 12-alpha-OH BAs, TDCA and GDCA, can activate TGR5 and stress kinases in hepatic stellate cells, thereby promoting cell proliferation and liver fibrosis. TDCA and GDCA treatment induces liver fibrosis and hepatic collagen deposition (92, 93). The effects of TDCA and GDCA extend beyond liver fibrosis. Correlations in humans show that TDCA and GDCA may be involved in insulin clearance and insulin resistance (94, 95). Higher levels of GDCA and DCA were also shown to be correlated with higher risk of T2D (93, 96, 97).

**Figure 5.**
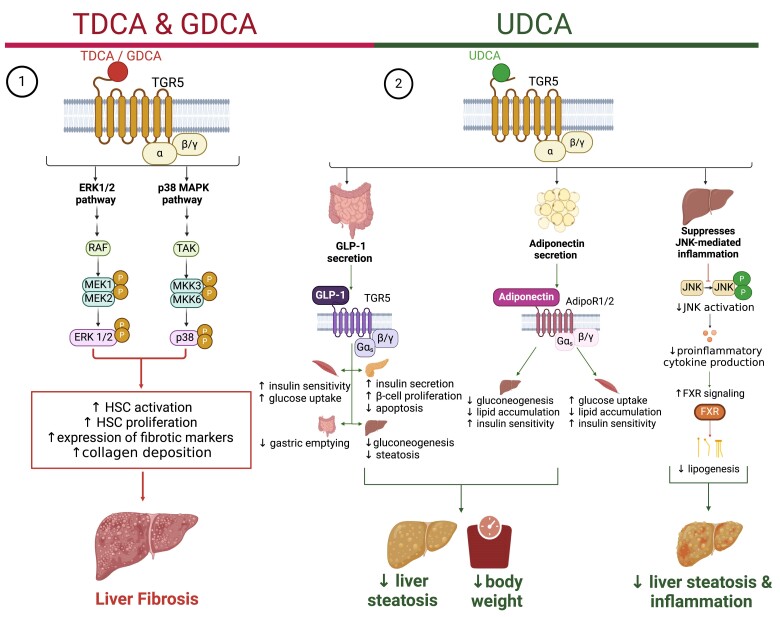
Secondary bile acids mediate host metabolism, inflammation, and fibrosis via TGR5 and FXR. (1) Conjugated secondary bile acids, TDCA & GDCA, activate ERK1/2 and p38 MAPK pathways through TGR5. Therefore, TDCA & GDCA signaling promotes expression of fibrotic markers, collagen deposition, and hepatic stellate cell activation and proliferation. (2) UDCA signaling through TGR5 promotes GLP-1 and adiponectin secretion from intestinal L-cells and adipocytes, respectively. UDCA signaling promotes insulin sensitivity and reduces lipid accumulation, which may reduce body weight and hepatic steatosis. UDCA also suppresses JNK activation, reducing proinflammatory cytokine levels, then lower inflammation mitigates suppression of FXR. Therefore, UDCA reduces hepatic inflammation and steatosis due to increased FXR activation, which lowers lipogenesis. Abbreviations: ERK 1/2, extracellular signal regulated kinase 1/2; FXR, farnesoid X receptor; GDCA, glycodeoxycholic acid; GLP-1, glucagon-like peptide 1; JNK, c-Jun N-terminal kinase; p38 MAPK, p38 mitogen-activated protein kinase; TDCA, taurodeoxycholic acid; TGR5, Takeda G-protein coupled receptor 5; UDCA, ursodeoxycholic acid. Created with BioRender.

In contrast to TDCA and GDCA, higher UDCA has shown to mitigate metabolic disease characteristics in mice (Fig. 5). UDCA is a secondary BA produced as a result of 7alpha/beta isomerization of chenodeoxycholic acid by bacterial enzymes (98). Oral administration of UDCA is an FDA-approved treatment for hepatobiliary diseases (98). Multiple studies have investigated its potential as a therapeutic for metabolic diseases, including MAFLD and obesity. Oral UDCA promoted weight loss and mitigated a similar cluster of metabolic disease characteristics in lean, obese, and aged mice, including lowering blood glucose, lipids, hepatic triglycerides, cholesterol, insulin resistance, and proinflammatory cytokines (98-100). Oral UDCA also improved liver steatosis and fibrosis in these mice by reducing hepatic fibrosis and inflammation (101). In mice, UDCA appears to work in a separate way compared to obeticholic acid (OCA), where UDCA may provide an alternative approach that avoids OCA-related side effects (102).

UDCA is a potent agonist of TGR5 and increases GLP-1 secretion from intestinal cells and adiponectin from adipocytes. UDCA-mediated TGR5 activation has also been shown to regulate immune responses, reducing hepatic inflammation (101). Proinflammatory cytokines such as TNF have been shown to downregulate FXR; therefore, the reduction of inflammation mediated by TGR5 signaling could result in increased FXR stimulation which regulates lipid metabolism (101). UDCA appears to improve gut barrier function, which could lower deleterious metabolic endotoxemia and inflammation; however, the evidence to date for meaningful changes to the microbiota by UDCA is limited to correlational changes in taxonomy (103). There are some differences in the actions of UDCA in mice and humans, since UDCA increases BA biosynthesis in mice. Mice tend to have an FXR antagonist BA pool with a major fraction being made up of beta-muricholic acid, a murine primary BA FXR antagonist. Therefore, alterations in the hepatic BA pool can dilute beta-muricholic acid levels (104, 105). This should be considered regarding potential postbiotic use in mice vs humans since oral UDCA administration can indirectly increase FXR activity in mice, by changing the balance of BA species, thereby promoting anti-lipogenic effects that may not translate well to humans (98). Oral UDCA administration to people with obesity and MAFLD antagonized FXR, which promoted lipogeneses in the liver, but importantly UDCA shifted the lipid accumulation of lipid species in liver (and adipose tissue) to neutral lipid species, which are potentially less metabolically deleterious (106, 107). This shift in lipid species could prevent lipotoxicity, where the UDCA-mediated shift to neutral lipids limits oxidative stress, inflammation, and metabolic disease pathogenesis in the liver (108-110).

Clinical trials of daily oral UDCA therapy via Ursoan^®^ in combination with exercise and caloric restriction have shown positive effects in MAFLD patients. including weight loss, reduction of liver disease markers (alanine aminotransferase [ALT], aspartate aminotransferase [AST], gamma-glutamyl transferase [GTT]), and serum lipids (triglycerides, total cholesterol [TC], low-density lipoprotein [LDL]). However, in these studies, no comparison was made between UDCA treatment alone vs in combination with lifestyle changes. Therefore, the exact role or contribution of UDCA to these effects cannot be determined (111, 112). In contrast, a clinical trial showed that oral UDCA (23-28 mg/kg/day) administration alone for 18 months showed no effect on histological improvement of MASH or reduction in weight, serum lipids, or markers of liver disease (113). Derivatives of specific BAs must be considered when assessing therapeutic potential or mechanisms of action of postbiotics. For example, higher isoUDCA, a derivative of UDCA, has been associated with higher appetite and higher postprandial blood lipids and inflammation. Bariatric surgery and fiber intake lowers isoUDCA, where levels of isoUDCA are determined by the gut microbiota (114). Therefore, it is important to consider how the microbiota modify potential BA interventions and how derivatives of BAs alter the net effect of BAs on host metabolism.

### Trimethylamine Oxide

Certain species of gut commensals metabolize nutrients such as choline and phosphatidylcholine in our foods to produce trimethylamine, which is absorbed and then converted into trimethylamine oxide (TMAO) by flavin-containing monooxygenase 3 in the liver (115). Increased TMAO levels are associated with changes in the composition of the gut microbiome (including a lower Bacteroidetes to Firmicutes ratio) that is characteristic of metabolic disease (116). The involvement of TMAO in cardiovascular disease has been widely studied, and more recently it has been investigated in the context of metabolic diseases, where higher TMAO levels are associated with higher risk of prediabetes and T2D (115, 117). Increased TMAO levels are associated with higher fasting levels of blood triglycerides and glucose, as well as higher HbA1c and insulin resistance (117-119). Dietary TMAO intake lowers transcripts involved in insulin signal transduction (*Akt, IRS2, PI3K*) and upregulates genes involved in glucose production via gluconeogenesis (*PEPCK, G6Pase*) in the liver (120).

TMAO levels are also associated with risk and severity of MASH in humans (121). Higher TMAO is associated with increased markers of liver injury (AST), steatosis, inflammation, ballooning, and overall MAFLD activity score in humans with T2D (121). Higher TMAO was also associated with higher triglycerides, cholesterol and LDL-cholesterol, and higher secondary BAs (DCA, GDCA, UDCA, and GLCA) (121, 122). Eighteen weeks of oral, dietary TMAO supplementation in a mouse model of MAFLD/MASH showed that TMAO skewed the BA pool to an FXR antagonism in the liver and serum and decreased FXR and small heterodimer partner protein expression (123). Consistent with TMAO lowering inhibition of lipogenesis by FXR, oral TMAO administration in these mice also exacerbated hallmarks of MAFLD, including higher triglycerides, liver to bodyweight ratio, steatosis, and liver injury markers (ALT, AST). TMAO also has a cell-autonomous effect on hepatocytes since TMAO increased fat deposition, shown by increased triglycerides and cholesterol, as well as promoted increased fibrotic gene expression in HepG2 cells (124). Overall, TMAO is involved in regulating lipid and glucose metabolism as well as liver fibrosis in T2D and MASH, but it is not yet clear how to target TMAO as a postbiotic. Theoretically, one potential way to lower TMAO would be to target trimethylamine production by the microbiota.

### D-Lactate

D-lactate is the chemical enantiomer of L-lactate that is involved in energy and carbon transfer through the Cori cycle (125). There are 2 endogenous sources of D-lactate: the methylglyoxal pathway and the gut microbiota. In the methylglyoxal pathway, glyoxylase 1 and 2 catalyze methylglyoxal into D-lactate (126). D-Lactate may act as a substrate for liver metabolism. It is broken down by D-lactate dehydrogenase or D-hydroxy acid dehydrogenase to produce pyruvate (127). Depending on the metabolic state of the organism, pyruvate can contribute to different metabolic pathways. In the fasted state, where blood glucose is lower, pyruvate is used as a substrate for gluconeogenesis (128). Pyruvate carboxylase metabolizes pyruvate into oxaloacetate which then can proceed to be converted into glucose (129). When blood glucose levels are higher, such as in the fed state, in the absence of dysmetabolism, pyruvate is used as a substrate for glycogen synthesis by conversion to glucose-1-phosphate and then glycogen formation through glycogen synthase (129). In conditions of glucose sufficiency, pyruvate is also a substrate for fatty acid synthesis (128). The pyruvate dehydrogenase complex produces acetyl-CoA from pyruvate which is then converted into malonyl-CoA by the activity of acetyl-CoA carboxylase 1 or 2. Malonyl-CoA is then metabolized by fatty acid synthase to produce palmitate (128, 130). Currently there is little published evidence for the concept of microbial-derived D-lactate fueling host metabolism, but it should be carefully considered, given that mammals have the enzymatic machinery to process D-lactate, and this postbiotic may be an alternate and underappreciated fuel for the Cori cycle.

In addition to host metabolism, D-lactate may also contribute to host immune function. MacDonald et al showed that the microbiota produce D-lactate in the gut lumen, which is a major source of D-lactate in the portal blood of mice (131). D-lactate can influence immune function in the intestine and liver, which is positioned to alter endocrine control of metabolism. Morita et al showed that gut microbial-derived D- and L-lactate promote antigen uptake by CX3CR1+ myeloid cells in the gut through activation of GPR31 (132). Therefore, bacterial-derived D-lactate promotes macrophage function and immune response in the intestinal lumen (132). D-lactate can activate immunity in Kupffer cells, resident macrophages in the liver, promoting their ability to capture and clear pathogens. MacDonald et al showed that Kupffer cells in mice with an intact microbiota infected intravenously with *Staphylococcus aureus* were able to eliminate the pathogen, whereas germ-free and antibiotic-treated mice were unable to mount a sufficient defense response to the pathogen (131). Importantly, Kupffer cell-mediated pathogen elimination in the absence of gut microbiota could be restored by administration of D-lactate in the gut or colonization with bacterial that produce D-lactate in mice (131). This demonstrates that microbial-derived D-lactate may act as an adjuvant to promote sufficient immunity in liver Kupffer cells during pathogen infection (131). Therefore, microbial-derived D-lactate is a postbiotic that participates in a gut-liver axis by promoting liver resident macrophage function. Hence, it is conceivable that microbial D-lactate promotes liver inflammation, insulin resistance, and metabolic dysfunction during obesity, prediabetes, T2D, and MALFD/MASH.

D-lactate levels are higher in blood and urine samples of people with diabetes (133) and rodent models of diabetes (134). Elevated blood glucose can fuel the methylglyoxal pathway; hence, it is not yet clear how much the microbiota contributes to elevated D-lactate levels in people with poorly controlled diabetes. However, people with MAFLD also have higher serum D-lactate, which is positively correlated with liver disease severity (135). Patients with moderate and severe hepatic steatosis had higher levels of D-lactate compared to people with mild steatosis (135). D-lactate programming of higher Kupffer cell-mediated inflammation is positioned to promote hepatic inflammation thereby promoting MAFLD/MASH progression (135-137).

**Figure 6.**
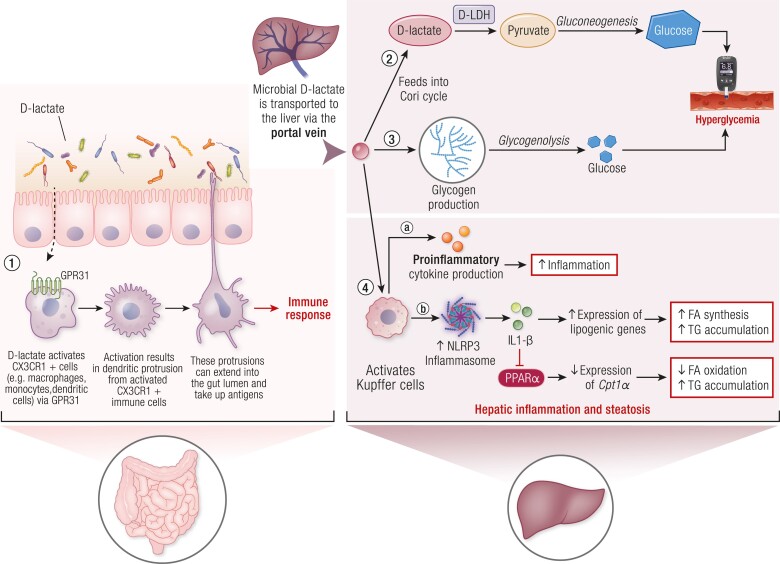
Microbial D-lactate contributes to host liver inflammation and metabolism. D-lactate produced by gut microbiota activates intestinal CX3CR1+ immune cells (1), including macrophages via GPR31 receptor, promoting antigen uptake. Microbial D-lactate is also transported to the liver through the portal vein where it may be used as a substrate for glucose production via the Cori cycle (2) and glycogen production (3). In the liver (4), D-lactate activates Kupffer cells to produce proinflammatory cytokines resulting in hepatic inflammation (4a). Kupffer cell-activation also activates NLRP3 inflammasome to produce IL-1β (4b). The resultant hepatic inflammation can upregulate expression of lipogenic genes and downregulate genes involved in FA oxidation by suppressing PPARα. Therefore, D-lactate-related hepatic inflammation is positioned to promote lipid accumulation in the liver. Abbreviations: CX3CR1, C-X3-C motif chemokine receptor 1; FA, fatty acid; GPR31, G-protein coupled receptor 31; IL-1β, interleukin 1-beta; KC, Kupffer cell; NLRP3, NOD-like receptor protein 3; PPARα, peroxisome proliferator-activated receptor alpha; TG, triglycerides.

D-lactate is also positioned to participate in blood glucose regulation. It is textbook knowledge that L-lactate derived from skeletal muscle glycolysis fuels the liver to produce glucose in the Cori cycle. Cori and Cori made these discoveries using D-lactate that was extracted from bacteria (to model fermented sarcolactic acid) (125). It was found that oral delivery or injection of D-lactate in rodents leads to glycogen deposition in the liver, whereas L-lactate delivery “hardly forms any liver glycogen” (125). These experiments concluded that “L-lactic acid is utilized [to form liver glycogen] 4 times more slowly [in rodents] compared to D-lactic acid” (125). Soffer et al have also shown that intravenous delivery of D-lactate to male human subjects resulted in the conversion of D-lactate into liver glycogen (138, 139). Recently, it was found that gut microbiota can regulate blood glucose exclusively through hepatic gluconeogenesis without microbiota-induced changes in energy expenditure or energy balance (140, 141). The bacterial metabolite or factor that acted as a substrate for changes in liver metabolism and hepatic gluconeogenesis remains unknown. Bacterial-derived D-lactate delivered to the liver through the portal vein may act as a key substrate contributing to liver glycogen and glucose. It is possible that bacterial D-lactate can feed into the Cori cycle via the portal vein and fuel hepatic production of glucose. Further investigation into the role of bacterial-derived D-lactate in hepatic metabolism and blood glucose is warranted because this potential new branch of the Cori cycle contains a carbon source outside the body. Hence, microbial D-lactate is a postbiotic that may be targeted in the gut to lower a substrate for host metabolism and/or lower liver inflammation (Fig. 6).

### Glycerol

Lipolysis is the major source of blood glycerol, where glycerol is liberated from triglycerides usually derived from adipose tissue, particularly in the fasted state (142, 143). Glycerol is then converted to glycerol-3-phosphate, which gets oxidized into dihydroxyacetone phosphate (DHAP). Depending on the metabolic needs of the cell/organism, DHAP can enter metabolic pathways such as glycolysis and gluconeogenesis, contributing to energy or glucose production (142, 144). Glycerol can also be converted to glycerol-3-phosphate to participate in lipogenesis. There are other sources of glycerol beyond adipose tissue lipolysis, including food and processes regulated by the gut microbiota.

Intestinal bacteria can generate changes in gut glycerol through the metabolism of triglycerides. Triglycerides are often not fully digested by host enzymes in the small intestine. Bacterial lipases, triacylglycerol acyl hydrolases, can hydrolyze glycerides into free fatty acids and glycerol. Bacterial phospholipases are also produced by the gut microbiota to hydrolyze glycerol from phospholipids (145). The specific hydrolase depends on the structure of the triglyceride, which is impacted by the length of fatty acid, degree of unsaturation, and position of fatty acid in the glycerol backbone. *Bacillus prodigiosus*, *B. pyocyaneus*, *B. fluorescens*, *Serratia marcescens*, *Pseudomonas aeruginosa,* and *Pseudomonas fluorescens* species are all lipase-producing bacterial strains (146). Lipase expression in bacteria is dependent on the presence of certain carbon sources, such as lipids, polysaccharides, and sugar alcohols (147). In general, a combination of glycerol, glycerides, and free fatty acids enter the colon, which has a higher abundance of bacteria. Therefore, the distal gut is positioned to contribute to glycerol liberation, but also glycerol genesis and metabolism. Gut-derived, microbially processed glycerol can then be transported to the liver through the portal vein to be further metabolized by entering glycolytic, gluconeogenetic, or lipogenic pathways (143).

Bacteria can also generate and metabolize glycerol (148). For example, *E. coli* uses glycerol-3-phosphate dehydrogenase and glycerol kinase to generate DHAP (149). In addition, bacteria such as *E. coli* can use an anaerobic fermentation pathway that includes glycerol dehydrogenase and dihydroxyacetone, producing nicotinamide adenine dinucleotide and phosphorylating phosphoenolpyruvate, generating DHAP. The fermentation pathway is favored in multiple types of bacteria including *E. coli*, *Pseudomonas*, and *Lactobacillus* (148, 150, 151). Once DHAP is generated, it can enter gluconeogenesis or glycolysis, and lead to the production of energy (149).

As a major contributor to gluconeogenesis, glycerol can influence blood glucose (152). The contribution of orally consumed glycerol to plasma glucose, via gluconeogenesis, was probed by ^13^C glycerol administration and was ∼20% lower in people with higher visceral adipose tissue, indicating that obesity is linked to a greater contribution of glycerol from endogenous adipose tissue, where this glycerol can contribute to hepatic gluconeogenesis (153). However, the methods used in this approach would not capture glycerol derived from the gut microbiota or microbiota-mediated glycerol liberation. It is not clear how obesity or metabolic disease alter the balance of glycerol production from adipose tissue vs gut-derived sources, and gut microbiota-derived glycerol may be an important substrate to consider beyond ingestion of glycerol.

**Figure 7.**
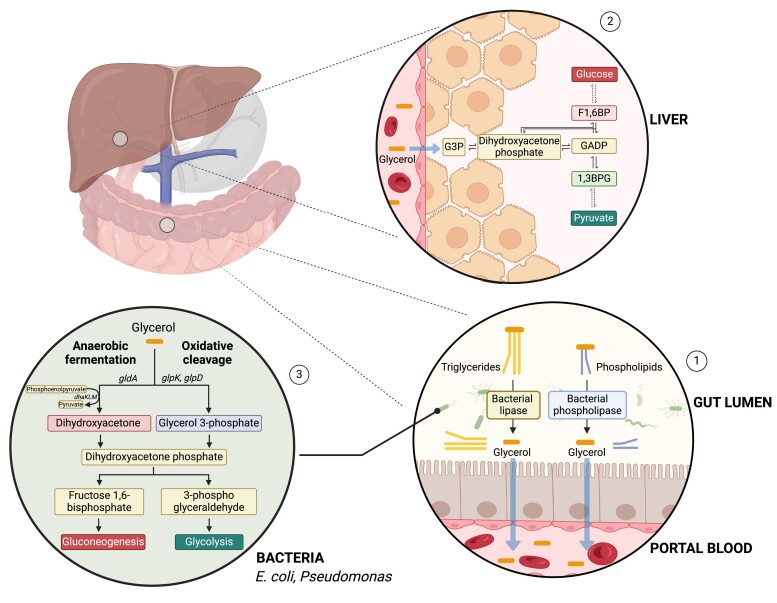
Microbiota processing of glycerol influences host metabolism. In the intestinal lumen (1), the bacterial lipases processes triglycerides from the host diet as well as phospholipids from bacterial and host membranes into free fatty acids and glycerol. Glycerol can translocate across cell membranes and can also reach the liver via the portal vein (via the gut-liver axis). In the liver (2), glycerol is converted into intermediates that participate in gluconeogenesis. Glycerol can also be metabolized by anaerobic fermentation or oxidative cleavage in bacteria such as *E. coli* and *Pseudomonas*. (3) In anaerobic fermentation, the action of the enzyme glycerol dehydrogenase (*gldA*) generates Dihydroxyacetone, while in oxidative cleavage the enzymes glycerol 3-phosphate dehydrogenase (*glpD*) and glycerol kinase (*glpK*) generate Glycerol 3-phosphate. Both are precursors of dihydroxyacetone phosphate which can be used in glycolysis or gluconeogenesis. Created with BioRender.

The impact of hepatic steatosis during MAFLD is similar to obesity, with lower orally administered glycerol contributing to plasma glucose (154). This work also observed a higher contribution of oral ^13^C glycerol to triglycerides in people with MAFLD potentiating accumulation of intrahepatic triglycerides (154). Therefore, the current dogma is that obesity and related metabolic diseases, such as MAFLD, skew glycerol metabolism toward adipose-derived glycerol, which has a higher lipogenic potential, and less reliance on oral glycerol (such as glycerol in food) to fuel gluconeogenesis and lipogenesis. However, it is possible that microbial-mediated glycerol production/liberation is higher in obesity and metabolic disease and is a significant contributor to host metabolism. Similar to D-lactate, gut-derived glycerol is a postbiotic that could be targeted to reduce substrate flux particularly relevant to hepatic gluconeogenesis and lipogenesis (Fig. 7).

### Succinate

Succinate is one of the intermediates of the tricarboxylic acid cycle (TCA) and the link between TCA and the mitochondrial respiratory chain in the host. However, *Bacteroides* spp., *Prevotella* spp., *Firmicutes* spp., and other microbiota bacteria are an important source of succinate through the fermentation of pentoses and hexoses (155). Succinate receptor 1 (SUCNR1; formerly known as GPR91) is expressed in immune cells, adipose tissue, liver, and kidneys (156-158). SUCNR1 can mediate immune responses, which positions succinate as a postbiotic that can have functions beyond serving as a metabolic intermediate.

Activation of SUCNR1 in immune cells (such as macrophages) favors induction of an anti-inflammatory profile through increased type 2 cytokines. In people with obesity, adipose tissue macrophages have low expression of SUCNR1, which is correlated with higher expression of the proinflammatory cytokines (159). People with obesity and T2D have higher levels of circulating succinate (160, 161). It has been postulated that an obesity-related increase in intestinal permeability accompanied by increased abundance of succinate-producing bacteria (Provotellaceae and Veillonellaceae) and reduction of succinate-consuming bacteria (Odoribacteraceae and Clostridaceae) is a mechanism that underpins a rise in blood succinate (161). Nevertheless, the source of higher succinate during obesity is not yet clear and it may be derived from host tissues. In fact, there is evidence that succinate in the gut does not necessarily leave the local gut environment. Increased intestinal succinate appears to be a beneficial postbiotic that acts locally.

The role of exogenous succinate, such as succinate derived from bacteria, has been linked to increased intestinal gluconeogenesis. Succinate levels can be modified by dietary supplementation in mice (with oral fructooligosaccharide) or colonization with *Prevotella copri*, which can increase succinate levels in the murine cecum, where succinate is used as substrate for intestinal gluconeogenesis. Increasing succinate in the gut does not increase succinate in the blood, showing that succinate is compartmentalized to the intestine for use in local gluconeogenesis (162). Succinate-induced intestinal gluconeogenesis increases glucose in the portal vein, which actually improves whole blood glucose homeostasis and decreases hepatic glucose production (162). A high-fiber diet is fermented by the microbiota into SCFAs and other metabolites such as succinate, which is the precursor for propionate. In the portal circulation, propionate acts as an agonist on free fatty acid 3/G-protein coupled receptor 41 (GPR41), signaling to the brain through the gut-brain axis to increase the release of vasoactive intestinal peptide and activating the expression of intestinal gluconeogenesis genes. Subsequently, both propionate and succinate, will serve as a substrate for intestinal gluconeogenesis through the TCA cycle (163). The compartmentalization of this process by a postbiotic is positioned to be beneficial for the host, as it is known that intestinal gluconeogenesis lowers hepatic steatosis, stimulation of thermogenesis in brown adipose tissue and browning process in the white adipose tissue and participates in endocrine control of metabolism, including control of insulin secretion (163-165). Future work could focus on how to manipulate succinate and amplify or restrict the actions of this postbiotic to act on receptors within the intestine, which then relays systemic metabolic benefits to the host.

### Ethanolamine

Enterobacteria can use phosphodiesterase to convert phosphatidylethanolamine into glycerol and ethanolamine. For many bacteria, ethanolamine is used as a source of carbon and nitrogen regulated by the *Eut* gene (166). Ethanolamine is higher in the intestine during obesity, which may, in part, be due to changes in the composition of the microbiota characteristic of obesity, which has lower expression of *Eut* genes and lower potential for metabolism of ethanolamine. The increase in intestinal ethanolamine can increase intestinal permeability through increased expression of miR-101a-3p which destabilizes the mRNA of zona occludens-1, a tight junction protein of the intestinal barrier (167, 168). A more permissive gut barrier caused by higher ethanolamine is conducive to inflammation and metabolic disorders through the translocation of bacterial components metabolites into circulation, including increased metabolic endotoxemia (7, 169). Additionally, diets of animal origin, rich in ethanolamine, also favor its accumulation in the intestine with a consequent increase in gut permeability (168). It is not yet clear how to target ethanolamine as a postbiotic beyond the usual approaches to manipulate ethanolamine producing and metabolizing commensal bacteria, which would have the limitations of probiotics or prebiotics. It may be difficult to target the precursor for ethanolamine since phosphatidylethanolamine can be derived from bacterial membranes, the diet, and even from the host. Targeting bacterial transport, metabolism and production pathways directly may be an option to manipulate gut levels of ethanolamine.

### Ethanol

Gut microbiota can generate ethanol through carbohydrate fermentation and metabolize ethanol through alcohol dehydrogenase, which converts ethanol into acetaldehyde (170). Ethanol can engage in a gut-liver axis to promote hepatic lipogenesis and inflammation. The metabolism of ethanol does not necessarily mitigate its detrimental effects on host metabolism. Even after ethanol is metabolized to acetaldehyde, this metabolite can increase intestinal permeability, which can promote metabolic endotoxemia and inflammation (171). It has been known for decades that endogenous ethanol production was possible in bacteria, but historically production of ethanol from commensal (resident) gut bacteria was not thought to be a significant source of host blood ethanol nor a contributor to liver disease (172). More recently, ethanol hyper-producing strains of *Klebsiella pneumoniae* have been isolated from the feces of individuals with severe MASH accompanied by auto-brewery syndrome. Germ-free mice colonized with strains of *Klebsiella pneumoniae* that produce excess ethanol have increased hepatic steatosis, mitochondrial dysfunction, and characteristics of MAFLD, effects that were potentiated by feeding an obesogenic diet (173). In addition, higher levels of ethanol were detected in the portal vein of people with MAFLD. When compared to levels in peripheral blood, ethanol levels in the portal vein were over 150 times higher, and ethanol level in the portal circulation correlated directly with the severity of steatohepatitis. Microbiota-derived ethanol in people with MAFLD was associated with increased intestinal lactic acid bacteria, especially *Streptococcus* and *Lactobacillus* species (174). It is not yet clear how to target bacterial-derived ethanol as a postbiotic. Targeting carbohydrate fermentation processes is one conceivable way in limiting bacterial production of ethanol. For example, *Weissella confusiona* has been described as an ethanol producer (in an individual presenting severe MASH), but in vitro experiments show that addition of other strains such as *Anaerostipes caccae*, *Lactobacillus acidophilus*, and *Lactobacillus fermentum* can limit ethanol production.

## Conclusions and Future Research Directions

Postbiotics are currently defined as “a preparation of inanimate microorganisms and/or their components that confers a health benefit on the host.” This allows the use of the term *postbiotic* when discussing preparations from whole bacteria or specific components of bacteria. It also allows the term *postbiotics* to be used in the presence or absence of bacterial metabolites (175). Postbiotics can cooperate or compete to influence host metabolism. Obesity and metabolic disease can skew the composition or compartmentalization of different postbiotics and tip the balance of microbial components and metabolites to promote metabolic dysfunction. Intervention at the level of postbiotics requires understanding of the microbial components and metabolites that are beneficial and deleterious for metabolic health and how they engage immune response that alter endocrine control of metabolism. Postbiotics with a given class, such as LPS, can have directly opposing effects on immunity and metabolism based on the structure-function relationship and engagement of host receptors. For example, hexa-acylated LPS from *E. coli* promotes insulin resistance, glucose intolerance, and metabolic inflammation, through activation of TLR4 (7). In contrast, under-acylated LPS is a TLR4 antagonist and can directly oppose dysmetabolism caused by hexa-acylated LPS and can even promote insulin sensitivity in obese mice (28). Thus, expanding the concept of postbiotics to include both beneficial effects and deleterious effects in the host is important.

It is known that the gut microbiota and microbial products such as postbiotics can interact with host endocrine system indirectly to alter hormone responses to catecholamines, leptin, and GLP-1 among other hormones relevant to metabolic disease (176-178). The concept of microbial endocrinology and the bidirectional relationship between host hormones and the gut microbiota have been expertly reviewed elsewhere (179, 180). There are many examples of the gut microbiome altering the regulation of gut hormone release, which has also been reviewed (181). The association of the gut microbiota with host endocrinology has typically been characterized through an indirect relationship, where hormone responses can influence bacterial composition and bacterial taxonomy or intermediates (such as SCFAs) are associated with altered endocrine status (180).

An important next step in postbiotic research will be to identify microbial molecules that can directly interact with the endocrine system and determine microbial peptides that mimic endocrine factors involved in metabolism. Recently, Girdhar et al discovered a gut microbial peptide that shares more than 50% of homology to an epitope in the B-chain of insulin (182). This insulin-like peptide is produced by the human gut commensal *Parabacteroides distasonis* and colonization with this bacterium leads to antibodies and autoimmune response that can accelerate onset of hyperglycemia in a mouse model of type 1 diabetes (T1D) (182). This study also showed an association between seroconversion relevant to T1D and presence of gut bacteria capable of producing the insulin mimic peptide (182). This suggests that a gut commensal can mimic insulin and trigger an immune response contributing to the development of T1D. Similarly, a microbial peptide that mimics islet-specific glucose-6-phosphatase catalytic subunit-related protein can activate CD8 T cells and promote the development of T1D in nonobese diabetic mice (183).

In addition, it was discovered that commensal *E. coli* can produce an antigen mimetic of alpha-melanocyte-stimulating hormone (alpha-MSH), which can regulate food intake and body mass in mice (184). *E. coli* also produces a melanocortin-like peptide that is similar to 2 mammalian melanocortin hormones, alpha-MSH and adrenocorticotropin (ACTH) (185). The melanocortin-like peptide of E. coli (MECO-1) was equally effective as alpha-MSH and ACTH in suppressing macrophage inflammation by acting on the melanocortin-1 receptor (MC1R). These highlight that bacterial peptides can have an endocrine effect in the host relevant to obesity and metabolic inflammation. However, the search for microbial-derived peptides that can mimic host endocrine factors should not be limited to bacteria. For example, mining of the viral genomes has already uncovered at least 16 viral homologues to human peptides, including insulin, insulin-like growth factors (IGF), fibroblast growth factors, adiponectin, and resistin (186). These factors have potential to act on host receptors, since chemical synthesis of viral insulin/IGF-1 peptides showed that these peptides can stimulate receptor signaling that is sufficient to increase adipocyte glucose uptake and lower blood glucose in mice (186). Specific viruses can also produce cytokines that can alter immune responses (187). An important future direction will be to determine the impact of newly discovered microbial hormone-like peptides and other microbiota-derived compounds that can mimic host factors and engage in endocrine or immune responses related to metabolic disease.

Microbiome research exploded with associations of taxonomy and disease. Prebiotics and probiotics often attempt to mitigate or correct changes in microbiome composition. It has been much harder to ascertain the functional units of the microbiome to provide evidence of cause-effect relationships and determine directionality in the host-microbe relationship. Postbiotics, microbial components and metabolites are one mechanism of action that can alter host metabolism, inflammation, and endocrine function. In this review, we summarized postbiotics derived from bacterial components or metabolites which can be either beneficial or detrimental to the host metabolism depending on which, where, and how they interact with the immune receptors or fuel host metabolism. Future work defining how postbiotics act as functional units of the microbiota to alter host metabolism is positioned to increase our understanding of the host-microbe relationship in metabolic health and disease.

## Abbreviations

ALT: alanine aminotransferase
AMPK: AMP-activated protein kinase
AST: aspartate aminotransferase
BA: bile acid
CD14: cluster of differentiation 14
DCA: deoxycholic acid
DHAP: dihydroxyacetone phosphate
FFAR2: free fatty acid receptor 2
FXR: farnesoid X receptor
GDCA: glycodeoxycholic acid
GLCA: glycolithocholic acid
GLP-1: glucagon-like peptide 1
HFD: high-fat diet
iE-DAP: gamma-D-glutamyl-meso-diaminopimelic acid
IL-: interleukin
IRS-1: insulin receptor substrate 1
LDL: low-density lipoprotein
LPS: lipopolysaccharide
LTA: lipoteichoic acid
MAFLD: metabolic dysfunction-associated fatty liver disease
MASH: metabolic dysfunction-associated steatohepatitis
MCP-1: monocyte chemoattractant protein-1
MDP: muramyl dipeptide
MSH: melanocyte-stimulating hormone
NF-κB: nuclear factor kappa B
NOD1/NOD2: nucleotide oligomerization domain protein ½
PYY: peptide YY
RIPK2: receptor-interacting serine/threonine-protein kinase 2
SCFA: short-chain fatty acid
SUCNR1: succinate receptor 1
T2D: type 2 diabetes
TCA: tricarboxylic acid cycle
TDCA: taurodeoxycholic acid
TGR5: G-protein coupled bile acid receptor 5
TLR: toll-like receptor
TMAO: trimethylamine oxide
TNF-α: tumor necrosis factor alpha
UDCA: ursodeoxycholic acid
